# The role of repeat fine needle aspiration in managing indeterminate thyroid nodules

**DOI:** 10.1186/s40463-019-0338-7

**Published:** 2019-03-20

**Authors:** Laura Allen, Ayham Al Afif, Matthew H. Rigby, Martin J. Bullock, Jonathan Trites, S. Mark Taylor, Robert D. Hart

**Affiliations:** 1 Faculty of Medicine, Dalhousie University, 1459 Oxford Street, Halifax, Nova Scotia B3H 4R2 Canada; 2 Dalhousie University Division of Otolaryngology – Head & Neck Surgery, QEII Health Sciences Centre, 3rd floor Dickson Building, 5820 University Avenue, Halifax, NS B3H 1Y9 Canada; 3 Department of Pathology, Dalhousie University, Sir Charles Tupper Medical Building, Room 11B, 5850 College Street, PO Box 15000, Halifax, NS B3H 4R2 Canada; 4 Department of Surgery, Section of Otolaryngology – Head and Neck Surgery, University of Calgary, 1007 North Tower, Foothills Medical Centre, 1403 29th Street NW, Calgary, AB T2N 2T9 Canada

**Keywords:** FNA, Thyroid, Bethesda system, Thyroid nodule

## Abstract

**Background:**

The Bethesda System is the most widely used for reporting fine needle aspiration (FNA) cytology. It recommends a repeat FNA (rFNA) when initial results are category I or III. It is unclear how often rFNA provides additional diagnostic information. We sought to investigate its utility at our institution.

**Methods:**

A retrospective chart review was performed of patients who had a category I or III FNA result and underwent rFNA of the same thyroid nodule between 2013 and 2015 at the QE II Health Sciences Centre in Nova Scotia, Canada. Results of initial FNA and ultrasound characteristics, rFNA, demographic data, surgical details, and pathology were collected.

**Results:**

A total of 237 patients (474 thyroid FNAs) were included. Most initial FNAs were category I (82%), the remainder category III (18%). rFNA yielded a different category 60% of the time. However, 60% remained category I or III. rFNA results of benign or malignant were found in 40% of cases; 1% were SFN/SFM. Twenty-seven percent of patients had surgery after rFNA; of those 68% had category I or III rFNA results. Of all nodules that underwent surgery, 46% were malignant, including 32% with category I rFNA results, and 42% category III.

**Conclusions:**

rFNA for category I and III nodules provided a definitive diagnosis in only 40% of cases, which is important for patient counseling. Malignancy rates at our centre were higher for these categories than predicted by Bethesda. Clinical management should consider institution specific malignancy rates, patient factors, and ultrasound findings.

## Background

Thyroid nodules are extremely common. Although the majority are benign, they warrant clinical evaluation to rule out malignancy. Thyroid cancer has the most rapidly increasing incidence rate among all cancers worldwide [1]. In developed countries, increased surveillance and the use of diagnostic technologies likely account for the rising number of early stage, asymptomatic thyroid cancers being diagnosed [1].

Fine needle aspiration (FNA) is a safe and cost effective tool used to evaluate thyroid nodules, and remains the world-wide gold standard for workup. FNA results are useful for stratifying malignancy risk and provide key information for counseling patients and determining the appropriateness of surgery. The Bethesda System for Reporting Thyroid Cytopathology is a 6-category, classification system created to standardize the interpretation of thyroid cytology [2]. Respectively, categories I-VI include: *non-diagnostic or unsatisfactory, benign, atypia of undetermined significance or follicular lesion of undetermined significance (A/FLUS), follicular neoplasm/suspicious for follicular neoplasm (SFN), suspicious for malignancy (SFM), and malignant.* This system provides category-specific malignancy rates and recommends appropriate clinical management for each category [2]. Nodules that have an initial FNA result of *non-diagnostic* or *A/FLUS* (category I or III, respectively) are recommended to undergo repeat FNA (rFNA) due to their associated malignancy risks, which are predicted by the Bethesda System to be 1–4% and 5–15%, respectively [2].

Studies on the utility of rFNA in providing additional diagnostic information for categories I and III nodules have yielded mixed results. Often, such nodules have been found to remain category I or III. Nodules with category I initial cytology have been reported to remain category I following rFNA in 17–47% of cases [3]. Similarly, nodules with an initial result of category III have been reported to remain category III or become category I in 19–31% and 1–7% of cases, respectively [3, 4]. Several studies have assessed the validity of rFNA following an initial diagnosis of category III [3–9]. It has also been suggested that core needle biopsy or immediate surgery are valid or superior to rFNA for the management of such nodules [3, 6–8]. Similarly, rates of malignancy following rFNA yield a wide range of results in the literature [6, 10]. A study by Vanderlaan et al. found that the use of rFNA following an initial diagnosis of category III was of minimal benefit as surgical pathology revealed similar rates of malignancy for patients who had rFNA and for those who underwent only a single FNA [6]. A recent retrospective chart review at a Canadian tertiary care centre found rFNA to be a valuable tool for further investigating category III nodules [5]. This study reported that 20% of patients with a category III initial FNA had a diagnosis of *SFM* or *malignant* following rFNA [5]. At our centre, 18.2% of category I nodules and 24.7% of category III nodules were found to be malignant in a 2013 study on malignancy rates by FNA category [11]. These rates are higher than those predicted by the Bethesda system [2]. Globally, few studies have shed light on rFNA following a category I initial FNA. Additionally, current evidence supports the use of ultrasound guidance to improve the diagnostic accuracy of thyroid masses [7, 8, 12–15]. A notable study by Yassa et al. in 2007 demonstrates the benefits of ultrasound guidance in the preoperative risk assessment for 2587 patients with imprecise FNA cytology [13].

The main goal of this study was to evaluate the utility of rFNA for clarifying the diagnosis in category I and III thyroid nodules. This was done by conducting an audit of FNA and rFNA results at our institution over a two-year period. Through this study, we aimed to add to the literature on our experience with the management of indeterminate FNAs, particularly with respect to the utility of rFNAs in our centre, and their role in clinical decision making.

## Methods

Ethics approval was obtained from the ethics board at our institution. The ethics board file number for this study is 1,021,250. We performed a retrospective chart review of male and female patients, 18 years and older, with an initial FNA result of Bethesda category I or III. All patients who underwent FNA and rFNA of the same nodule between 2013 and 2015 were included in the study. All FNAs and rFNAs were reviewed by cytopathologists at the QEII Health Sciences Centre. A total of 237 patients were included, and a total of 474 FNA/rFNAs. All FNA/rFNAs were ultrasound-guided with the exception of 6 patients. Patients who had initial and rFNAs performed on separate nodules were excluded. Patient demographic data, whether surgery was undertaken, the type and extent of surgery, and post-operative pathology reports were also obtained from medical records. Characteristics of the lesion described on ultrasound including size, vascularity, echogenicity, and associated calcifications were also collected for initial FNAs. Data were tabulated, analyzed, and figures were made using Microsoft Excel version 15.33.

## Results

### Patient and nodule demographics

A total of 237 patients with category I or III FNA cytology underwent rFNA at the Queen Elizabeth II Health Sciences Centre between 2013 and 2015. The mean age at diagnosis was 55.8, with a range of 22–87 years. In total, 189/237 (79.7%) were female, and 48/237 were male (20.1%; Table 1). The average time from initial to rFNA was 3.4 months.

**Table 1:** Patient demographics

Variable	Value
Age, years	
Mean (years)	55.8
Range (years)	22–87
Sex	
Male % (n)	20.3 (48)
Female % (n)	79.7 (189)
Size of Lesion	
1-2 cm	64
2-4 cm	119
> 4 cm	52
Unspecified	2

### FNA and post-operative pathology results

Most nodules (82%) had an initial FNA result of category I while the remainder had an initial result of category III (18%). Most nodules (60%) remained within the same categories (I or III) after rFNA (Table 2).

**Table 2:** Proportion of rFNA results as a subset of initial FNA results, and percentage of patients undergoing surgery in each group

Initial FNA	% (*n*)	Repeat FNA result	% of Initial (*n*)	% Surgery (*n*)	% Malig. (*n*)
*Non-diagnostic*	82 (194)	*Non-diagnostic*	36 (69)	25 (17)	29 (5)
		*Benign*	37 (71)	11 (8)	63 (5)
		*A/FLUS*	24 (47)	45 (21)	43 (9)
		*SFN/SFM*	1 (3)	100 (3)	33 (1)
		*Malignant*	2 (4)	100 (4)	75 (3)
*A/FLUS*	18 (43)	*Non-diagnostic*	21 (9)	22 (2)	50 (1)
		*Benign*	30 (13)	8 (1)	0 (0)
		*A/FLUS*	40 (17)	18 (3)	33 (1)
		*SFN/SFM*	0 (0)	0 (0)	0 (0)
		*Malignant*	9 (4)	100 (4)	100 (4)

*A/FLUS* – atypia/follicular lesion of unknown significance; *SFN/SFM* (suspicious for neoplasm/suspicious for malignancy)

Of all patients with an initial category I FNA, 36% remained within the same category following rFNA, 37% became *benign*, 24% became *A/FLUS*. Only 2% became either *SFN/SFM* or *malignant,* respectively. For patients in the abovementioned rFNA groups, 25, 11, 45, 100 and 100% underwent surgery, respectively. For patients with category I initial and rFNA results who underwent surgery, 29% were found to have malignancy. For patients with category I initial FNA results who underwent surgery following an rFNA result of category III, 43% were found to have malignancy (Table 2).

Of patients with an initial category III FNA, 40% remained within the same category following rFNA, while 21% became *non-diagnostic* and 30% became *benign*. None of the rFNAs yielded a *SFN/SFM* result, and only 9% became *malignant*. In the abovementioned rFNA groups, 22, 8, 18, 0 and 100% underwent surgery, respectively. For patients with category III initial and rFNA who underwent surgery, 33% were found to have malignant post-operative pathology. For patients with category III initial FNA results who underwent surgery following an rFNA result of category I, 50% had malignant post-operative pathology (Table 2).

Overall, of all patients who had rFNA, 27% underwent surgery. Thus, post-operative pathology malignancy rates for nodules that remained categories I and III following rFNA ranged from 29 to 50% (Table 2).

### Ultrasound features

Most patients (98%) underwent ultrasound guided initial FNA. Amongst those with an initial category I FNA, 44% were described as solid or with solid component(s), 32% as cystic or with a cystic component, 23% as hypoechoic, 8% as hyperechoic, 29% as hypervascular, 18% with microcalcifications, and 15% as complex. A similar distribution was seen in patients with category III FNAs (Table 3).

**Table 3:** Ultrasound characteristics of nodules by initial FNA result

Lesion characteristic	Category I FNA % of total (*n*)	Category III FNA % of total (*n*)
Solid/solid component	44 (86)	51 (22)
Cystic/cystic component	32 (62)	23 (10)
Hypoechoic	23 (44)	16 (7)
Hyperechoic	8 (16)	5 (2)
Hypervascularity	29 (56)	14 (6)
Microcalcification(s)	18 (34)	21 (9)
Complex nodule	15 (30)	2 (1)

## Discussion

This study investigated the value of rFNA following category I or III initial FNA cytology for thyroid nodules in a Canadian tertiary care centre. rFNA resulted in a different FNA category in approximately 60% of cases. However, the majority (60%) of nodules remained category I or III following rFNA. rFNA resulted in a *benign, SFN/SFM,* or *malignant* category in approximately 40% of cases. Specifically, of the changed (40%) category I nodules on rFNA; 95% became *benign*, 1% *SFN/SFM,* and 4% *malignant*. Of the changed category III nodules on rFNA; 75% became *benign,* and 25% became *malignant*.

The management of category I and III nodules can be rather challenging. The American Thyroid Association (ATA) advocates that in the presence of worrisome clinical or radiographic features; rFNA, or surgical excision are valid management options. Additionally, molecular testing has been suggested to provide a diagnostic benefit for patients with category III results, although the long-term outcomes remain unknown. We offer molecular testing to all patients with category I and III nodules. However, this option is seldom taken due to the cost, which is not covered in our health jurisdiction. In the event of initial category I cytology, rFNA is initially recommended. For multiple category I rFNAs, or worrisome clinical or radiographic features, surgery has been proposed as a valid option [16].

Few studies have looked at the clinical utility of rFNA following category I or III cytology. Category I cytology has been reported to remain category I following rFNA in 17–47% of cases [3]. Our data were within this range, with these nodules remaining category I 36% of the time and becoming category III 24% of the time after rFNA. Category III nodules have been reported to remain category III or become category I in 19–31% and 1–7% of cases after rFNA, respectively [3, 4]. Our study found higher rates, with 40% of category III nodules having remained category III following rFNA and 21% becoming category I.

In this study, nodules with category I or III initial cytology had a relatively high likelihood of remaining category I or III after rFNA (60%). Only 40% of rFNAs yielded a *benign, SFN/SFM,* or *malignant* category. Of those, 88% were *benign*, 3% *SFN/SFM*, and 8% *malignant*. Thus, we found that rFNA of category I and III nodules often remained *non-diagnostic/unsatisfactory* or *A/FLUS*, with definitive rFNA results of *benign* being more frequent than *malignant*. This is lower than reported in a retrospective chart review of 730 patients that found rFNA for category III nodules yielded definitive results at a rate of 65% [17]. By contrast, a recent Canadian study by *Jooya* et al. looked at rFNA for category III and *SFN* nodules and found 76% of patients remained within the same categories. However, 20% of patients with a category III result were then found to be *SFM* or *malignant* following rFNA, which is higher than in our study where 9% of category III nodules were malignant following rFNA [5].

Overall, 46% of patients who underwent surgery after rFNA were found to have malignant post-operative pathology. Forty-three percent (*n* = 12/28) of nodules with PTC surgical pathology were incidental findings of micro PTC. This percentage changed to 37% when accounting for only lesions with category I and III pre-operative rFNA results. In this study, 27% of patients with an initial category I FNA had surgery following rFNA. Of those, 44% were found to have malignant post-operative pathology. Of patients with an initial category III FNA, 23% went on to have surgery following rFNA, and of those 60% had malignant surgical pathology. It is important to place these findings into a centre-specific context. At our centre, categories I and III comprise 48% of all initial thyroid FNAs [11]. Thus, the rate of category I and III FNAs is higher at our centre than data published in Bethesda [2]. Additionally, in our centre, 18.2% of category I nodules, and 24.7% of category III nodules were found to be malignant, which is higher than the malignancy rates predicted by the Bethesda system [11]. Knowledge of such data is essential for patient counseling and for managing patient expectations following rFNA. It is our recommendation that patient factors, ultrasound features of the nodule(s), FNA results and centre-specific rates of malignancy per FNA category be used in decision-making regarding surgery. Based on our institutional data, offering surgery to patients with category I or III nodules, without an rFNA, is a valid approach in the presence of worrisome features on ultrasound or history.

The vast majority of patients in this study underwent ultrasound guided FNA. In the presence of multinodular disease, this adds to the accuracy of our findings. The most recent ATA Guidelines emphasized the importance of ultrasound features in predicting the risk of malignancy in thyroid nodules. In this study, category I and III nodules had a similar proportion of high risk features, such as microcalcifications and hypoechogenicity.

Like any study however, the study at hand has limitations. The data are retrospective, and represented patients from a single institution, and represent a small sample size. Multicenter data are essential for drawing broader and more generalizable conclusions. The FNAs were collected by multiple radiologists, and interpreted by several pathologists at our centre, which may create inconsistency in sample collection and interpretation. The vast majority of indeterminate FNA results were category I in this study. We have previously published on the high rate of category I FNAs in our centre [11] when compared to other studies, including the Bethesda study [2]. This may be due to shortcomings in how samples are collected and preserved in our centre. Additionally, thyroid FNAs are interpreted by multiple cytopathologists, in contrast to larger centres with a dedicated pathologist. This further speaks to the heterogeneity in reporting thyroid FNAs between centres. In our study, rFNA was performed 3.4 months, on average, after the initial FNA. It was previously suggested that performing an rFNA at a short interval after initial FNA may lead to higher rates of false positive. However, recent studies found no difference in the accuracy of rFNA whether performed 3 or 6 months after initial FNA [18, 19].

## Conclusions

rFNA plays a role in clinical decision making for patients with an initial FNA of category I or III and has been found to re-classify nodules to *benign, malignant,* or *SFN/SFM* in 40% of cases. Importantly, however, 60% of nodules remained category I or III following rFNA. Approximately one quarter of patients with an initial category I or III FNA who then underwent rFNA went on to have surgery, and 46% of these patients were found to have malignant post-operative pathology. Our centre has higher rates of category I and III FNAs, and the malignancy rates within these categories was also higher than predicted by the Bethesda system [11]. We recommend not relying solely on FNA cytology. It is important to consider centre-specific malignancy rates, as well as ultrasound characteristics and individual patient factors in a broader context to guide clinical decision making. Based on our data, offering surgery to patients with category I or III nodules on initial FNA, without an rFNA, is a valid approach in the presence of worrisome features.

## Abbreviations

A/FLUS: atypia/follicular lesions of unknown significance
ATA: American Thyroid Association
FNA: fine needle aspiration
rFNA: repeat fine needle aspiration
SFM: suspicious for malignancy
SFN: suspicious for follicular neoplasm
